# Supplementary material for: Exploring behaviours perceived as important for human—Dog bonding and their translation to a robotic platform
Source: PLoS One. 2022 Sep 28;17(9):e0274353. doi: 10.1371/journal.pone.0274353 (PMC9518860; doi:10.1371/journal.pone.0274353)
Supplement: S1 Table — (PDF) [file pone.0274353.s001.pdf]

S1 Table - Prominent Themes Identified by Coders 1 and 2

Coder 1 (Full Dataset)	Coder 2 (20% Subset, Randomly Allocated)
Physical contact/Close Proximity<br>● Spontaneous touching and cuddling<br>● Monitoring<br>● Following	Physical affection initiation and responds to affection and need for closeness, physical contact and touch<br><br>Desires proximity and following behaviour
Positivity and Enthusiasm<br>● Importance of Greeting enthusiasm<br>● Consistency of Positivity	Positive emotional expression, greetings, show appreciation
Perceived intelligence/higher level thinking<br>● Perceived Emotional Intelligence<br>● Communicative eye gaze and object-bringing<br>● Responsive to words or gestures	Attuned to and responsive to human emotions and daily life routine<br><br>Communication - communicates needs to owner - respond to owner commands - bring toys and gifts<br><br>Protective behaviour, safe-base, and checking in behaviour
Time spent together<br>● Importance of Play<br>● Perceived mutual enjoyment	Playful behaviours, games, initiates play<br><br>Shared mutual activities and shared enjoyment includes physical such as exercise and training
Sleeping behaviour	Sleeping behaviour
Relationship to the owner<br>● Love mentioned<br>● Part of the family<br>● Student-teacher dynamic	
Perceptions of dog as:<br>● Simple, innocent, or child-like<br>● Independent<br>● Intentional<br>● In tune or synchronous<br>● Protective	
Benefits to the owner<br>● Comfort during sadness<br>● Mental Health<br>● Fitness/Physical activity	
