# Supplementary material for: Exploring behaviours perceived as important for human—Dog bonding and their translation to a robotic platform
Source: PLoS One. 2022 Sep 28;17(9):e0274353. doi: 10.1371/journal.pone.0274353 (PMC9518860; doi:10.1371/journal.pone.0274353)
Supplement: S2 Table — (PDF) [file pone.0274353.s002.pdf]

## S2 Table – Key Words within each Theme

| Theme | Sub-themes | Key Words |
|---|---|---|
| Attunement | Daily Routine | Day<br>Routine<br>Same<br>Sleep |
| | Emotional | Emotion<br>Feeling<br>Sad<br>Upset<br>Comfort<br>Negative |
| | Other | Sync<br>Same<br>Shared |
| Communication | Communicative Eye Gaze | Eye<br>Gaze<br>Look |
| | Expressing Needs | Need<br>Want<br>Noise<br>Toy<br>Object |
| | Responsive to Word or Gesture | Responds<br>Word<br>Say<br>Gesture<br>Move |
| Consistency and Predictability | Consistent | Always<br>Usually |
| | Inconsistent | Sometimes<br>Maybe |
| Perceptions of the human | Simplicity or Innocence | Simple<br>Innocent<br>Child<br>Pure |
| | Dog as family | Family<br>Unit |
| | General Intelligence | Smart<br>Intelligent<br>Clever<br>Know |
| | Mutuality | Mutual<br>Both<br>Us |
| | Behaviours as Intentional | Intention<br>Purpose |
| Physical Affection | Responsive Touch | Touch<br>Nudge<br>Nuzzle<br>Push |
| | Spontaneous Initiation | Spontaneous<br>Decides<br>Ask |
| Positivity and Enthusiasm | Importance of Greeting Enthusiasm | Greet<br>Home |
| Proximity | Monitoring | Monitor<br>Check |

| | | |
|---|---|---|
| | Physical Following | Follow<br>Shadow |
| | Sleeping Behaviours | Sleep<br>Nap |
| Shared Activities | Importance of Play | Play<br>Fun |
| | Walks | Walk |
| | Other Activities | Teach<br>Train<br>Sport<br>Activity<br>Together |
