# Supplementary material for: Exploring behaviours perceived as important for human—Dog bonding and their translation to a robotic platform
Source: PLoS One. 2022 Sep 28;17(9):e0274353. doi: 10.1371/journal.pone.0274353 (PMC9518860; doi:10.1371/journal.pone.0274353)
Supplement: S1 File — (PDF) [file pone.0274353.s003.pdf]

Lexington Attachment to Pets Scale [25]

1

Please tell us whether you agree or disagree with some very brief statements about your favourite pet.

	Strongly agree	Somewhat agree	Don't know/ refuse to answer	Somewhat disagree	Strongly disagree
Quite often I confide in my pet.	○	○	○	○	○
I believe that pets should have the same rights and privileges as family members.	○	○	○	○	○
I believe my pet is my best friend.	○	○	○	○	○
Quite often, my feelings toward people are affected by the way they react to my pet.	○	○	○	○	○
I love my pet because he/she is more loyal to me than most of the people in my life.	○	○	○	○	○
I enjoy showing other people pictures of my pet.	○	○	○	○	○
I think my pet is just a pet.	○	○	○	○	○
I love my pet because it never judges me	○	○	○	○	○
My pet means more to me than any of my friends.	○	○	○	○	○
My pet knows when I'm feeling bad.	○	○	○	○	○
I often talk to other people about my pet.	○	○	○	○	○
My pet understands me.	○	○	○	○	○
I believe that loving my pet helps me stay healthy.	○	○	○	○	○
Pets deserve as much respect as humans do.	○	○	○	○	○
My pet and I have a very close relationship.	○	○	○	○	○
I would do almost anything to take care of my pet.	○	○	○	○	○
I play with my pet quite often.	○	○	○	○	○
I consider my pet to be a great companion.	○	○	○	○	○
My pet makes me feel happy.	○	○	○	○	○
I feel that my pet is a part of my family.	○	○	○	○	○
I am not very attached to my pet.	○	○	○	○	○
Owning a pet adds to my happiness.	○	○	○	○	○
I consider my pet to be a friend	○	○	○	○	○
