# Supplementary material for: Exploring behaviours perceived as important for human—Dog bonding and their translation to a robotic platform
Source: PLoS One. 2022 Sep 28;17(9):e0274353. doi: 10.1371/journal.pone.0274353 (PMC9518860; doi:10.1371/journal.pone.0274353)
Supplement: S2 File — (PDF) [file pone.0274353.s004.pdf]

<u>Demographic Questions</u>

All questions below were open - with responses collected using ‘free text’ boxes:

- What is your age?
- Please state your gender.
- How old is your dog? (Please state the answer in numbers only e.g. 1 year 6 months = 1.5)
- What sex is your dog?
- What breed is your dog?
- How long have you owned your dog? (Please state the answer in numbers only e.g. 1 year 6 months = 1.5)
- Do you have any other pets? If so, state species and how long owned for.

The following questions were closed - using multiple choice options:

- How did you acquire your dog?
    - Breeder
    - Rescue
    - Friend/Family Member
    - Other (please specify below)
- If you selected "Other", please specify:
    - [free text box]
